# Maintenance Immunosuppression in Kidney Transplantation: A Review of the Current Status and Future Directions

**DOI:** 10.3390/jcm14061821

**Published:** 2025-03-08

**Authors:** Muhammad Ali Khan, Alessandra Hanna, Srilekha Sridhara, Harshad Chaudhari, Hay Me Me, Rose Mary Attieh, Bassam G. Abu Jawdeh

**Affiliations:** 1 Division of Nephrology and Hypertension, Mayo Clinic Arizona, 5777 E. Mayo Blvd, Phoenix, AZ 85054, USA; hanna.alessandra@mayo.edu (A.H.); sridhara.srilekha@mayo.edu (S.S.); chaudhari.harshad@mayo.edu (H.C.); me.hayme@mayo.edu (H.M.M.); 2 Department of Transplant, Mayo Clinic, Jacksonville, FL 32224, USA; attieh.rosemary@mayo.edu

**Keywords:** immunosuppression, belatacept, calcineurin inhibitors, mycophenolic acid, kidney allograft

## Abstract

Kidney transplantation remains the gold standard for managing end-stage kidney disease, providing superior survival and quality-of-life outcomes compared to dialysis. Despite the ongoing gap between organ availability and demand, it is inevitable that kidney transplantation will continue to grow. This is owed to broader organ sharing, increased comfort of transplant programs with marginal kidney utilization, and the expansion of paired exchange among living donor kidneys. The evolution of kidney transplantation could not have been possible without the availability of effective immunosuppressive regimens that prevent rejection and maintain graft function. Mycophenolic acid and calcineurin inhibitors continue to serve as the foundation of modern maintenance immunosuppression. While these agents have markedly reduced acute rejection rates, their long-term efficacy in graft survival remains suboptimal. Alternative immunosuppressive therapies, including belatacept and mammalian target of rapamycin inhibitors, have demonstrated potential benefits. However, concerns regarding an increased risk of rejection have limited their widespread adoption as primary treatment options. In addition to ongoing efforts to refine steroid- and calcineurin inhibitor-sparing strategies, the identification of practical and quantifiable biomarkers for predicting long-term graft survival remains a critical objective. This review evaluates contemporary immunosuppressive protocols, highlights existing challenges, and explores future directions for optimizing long-term transplant outcomes.

## 1. Introduction

Transplantation has transformed the management of end-stage organ failure, offering life-saving solutions to patients who would otherwise have few or no options. For individuals with end-stage kidney disease (ESKD), kidney transplantation provides an opportunity to improve patient survival and quality of life compared to dialysis [1,2,3].

The first successful kidney transplant was performed between identical twins in December 1954 by Dr. Joseph Murray and his colleagues and marked a milestone in medical history [4]. While advances in organ preservation and surgical techniques have been pivotal to transplantation success [5], the management of recipient immunity remains the most critical factor for ensuring favorable outcomes. As a result, the widescale increase in the number of kidney transplants did not pick up until three decades later. The 1980s ushered in a significant era in transplantation with the introduction of the calcineurin inhibitor (CnI), cyclosporine [6,7]. The availability of anti-metabolite and CnI-based immunosuppression (IS) significantly improved acute rejection rates and therefore short-term outcomes. Despite this advancement, long-term graft survival improvement has remained limited, partially due to the nephrotoxicity and other metabolic adverse effects associated with IS. Prolonged exposure to CnI is associated with nephrotoxicity including arteriolar hyalinosis, tubular injury and interstitial fibrosis [8,9]. Moreover, steroids often used in maintenance IS regimens have been associated with cardiovascular events and mortality. To improve longer term outcomes, efforts have more recently focused on examining CnI- and steroid-sparing regimens. In this review, we explore contemporary maintenance IS regimens and their associated adverse effects. We also highlight recent advances in balancing efficacy and long-term safety, shedding light on modern approaches to improving outcomes in kidney transplantation. By examining progress and persisting challenges, this review underscores the field’s enduring pursuit of better patient care and outcomes.

## 2. T-Lymphocyte Activation and Site of Action of Immunosuppressive Drugs

**Figure 1:**
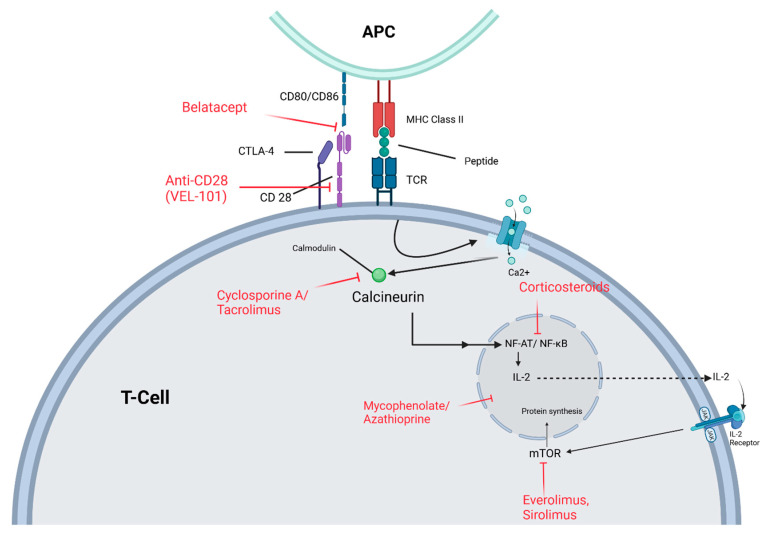
T-cell Activation: T-cell activation begins when the TCR binds to an antigen presented by an APC via MHC class II. Co-stimulation through CD28 and CD80/CD86 enhances activation. Upon TCR engagement, intracellular calcium influx activates calcineurin, which dephosphorylates NFAT, enabling it to enter the nucleus and drive IL-2 transcription. IL-2 promotes T-cell proliferation via the mTOR pathway. Immunosuppressive drugs act at different steps: calcineurin inhibitors (cyclosporine/tacrolimus) block NFAT activation and IL-2 production, mTOR inhibitors (everolimus/sirolimus) prevent IL-2-driven proliferation, antimetabolites (mycophenolate/azathioprine) disrupt DNA synthesis, corticosteroids suppress cytokine production, and co-stimulation blockers (belatacept, anti-CD28 VEL-101) prevent early activation by inhibiting the CD28-CD80/CD86 interaction. Abbreviations: APC: Antigen-Presenting Cell; Ca^2+^: Calcium Ion; CD28: Cluster of Differentiation 28; CD80/CD86: Cluster of Differentiation 80 / Cluster of Differentiation 86; CTLA-4: Cytotoxic T-Lymphocyte-Associated Protein 4; IL-2: Interleukin-2; mTOR: Mammalian Target of Rapamycin; MHC Class II: Major Histocompatibility Complex Class II; NF-AT: Nuclear Factor of Activated T-cells; NF-κB: Nuclear Factor Kappa-Light-Chain-Enhancer of Activated B Cells; TCR: T-Cell Receptor. (Created in BioRender. Khan, M. (2025) https://BioRender.com/n21u240 accessed on 30 January 2025).

To understand the mechanism of action of current immunosuppressive medications, it is crucial to understand the T-lymphocyte (T-cell) activation cascade (Figure 1). T-cells are pivotal for the immune system and are therefore responsible for transplant organ rejection.

The regulation of T-cell activation is delicately balanced between positive and negative signals, a dynamic that is crucial in preventing transplant rejection and promoting tolerance. This process necessitates two important signals:

- Antigen recognition: The first positive signal occurs when an antigen-presenting cell (APC) presents a foreign antigen (donor kidney antigen) to T-cells via major histocompatibility complex (MHC) molecules [10]. This interaction forms a weak bond that primes the T-cell for further activation.
- Co-stimulatory signal: The second signal involves co-stimulatory molecules that fully activate the T-cell. This signal is vital for preventing T-cell tolerance. Without it, a T-cell can enter a state of anergy [11], rendering it unresponsive to future exposure to the same antigen. Co-stimulatory interactions, such as the binding of CD28 on the T-cell to CD80 (B7-1)/CD86 (B7-2) on the APC, are key to this process.

Following complete T-cell activation, a cascade of intracellular events is triggered. The initial event is a calcium influx that activates the calmodulin pathway, ultimately leading to the activation of calcineurin. Calcineurin dephosphorylates nuclear factor of activated T-cells (NFAT) family proteins, promoting their translocation into the nucleus. Once inside the nucleus, NFAT activates the transcription of key genes, particularly interleukin-2 (IL-2), which is critical for T-cell proliferation and differentiation, acting as an autocrine signal. Disruption of this process results in the cessation of IL-2 production, thereby inhibiting T-cell differentiation and proliferation [12].

As IL-2 is produced and secreted, the T-cell enhances the expression of IL-2 receptor (CD25) on its surface. The secreted IL-2 binds to its receptors in an autocrine fashion, initiating another intracellular signaling pathway. This kinase-dependent cascade involves the mammalian target of rapamycin (mTOR), a protein that, when fully activated, promotes the translation of new proteins necessary for cell growth. Consequently, the T-cell progresses from the G1 phase to the S phase of the cell cycle, allowing it to grow and proliferate [13].

Immunosuppressive medications prevent rejection by mechanisms targeting the various stages of the T-cell response and activation [14] (Figure 1 and Table 1).

## 3. Current Maintenance Immunosuppression Regimens

***Anti-metabolite and CnI-based regimens:*** Mycophenolic acid and tacrolimus comprise the backbone of modern IS [48]. The landmark trial which perhaps led to solidifying this practice is the ELITE Symphony trial [49]. In this study, kidney transplant recipients (KTRs) were randomly assigned to either a regimen of standard-dose cyclosporine, mycophenolate mofetil, and glucocorticoids or a regimen of daclizumab induction, mycophenolate mofetil, and glucocorticoids in combination with low-dose cyclosporine, low-dose tacrolimus, or low-dose sirolimus. The group receiving low-dose tacrolimus showed the most favorable results, including a reduction in acute rejection by more than half and better graft survival.

In a study by Davis et al. the authors attempted to identify the desired tacrolimus trough level [50]. They found that a mean tacrolimus trough level of <8 ng/mL was linked with de novo donor specific antibody (dnDSA) at 1 year with an odds ratio of 2.32. Moreover, this translated into an increased acute rejection rate and 5-year death-censored graft loss [50]. This is in keeping with previous studies showing that nonadherence with CnI is a risk factor for dnDSA and graft fibrosis [51]. Based on these data, a reasonable long-term tacrolimus trough would be around 6–8 ng/mL [52]. Higher levels may predispose KTRs to adverse events and long-term nephrotoxicity.

The current antimetabolite of choice for transplant centers remains to be mycophenolic acid. In a meta-analysis of 19 studies, mycophenolic acid combined with CnI reduced the risk of acute rejection compared to azathioprine-CnI based IS (RR 0.62) [53]. This benefit from mycophenolic acid was observed irrespective of the type of CnI used. Moreover, patients receiving mycophenolic acid had a lower risk of graft loss (RR 0.76). The OPTICEPT trial compared fixed versus concentration-controlled mycophenolic acid dosing. The fixed dose arm received 2g per day versus titrating the dose to a target mycophenolic acid level of ≥1.3–1.9 mcg/mL (depending on whether the patients were on cyclosporine or tacrolimus) [54]. The glomerular filtration rate (GFR) and acute rejection in the concentration-controlled arm were not inferior to standard dosing; however, concentration-controlled patients required higher doses on average. Current practice for most centers is to use fixed mycophenolic acid dosing and titrate based on adverse events.

***Early steroid withdrawal immunosuppression regimens:*** Early steroid withdrawal (ESW) regimens have been sought to minimize the metabolic side effects commonly associated with glucocorticoids. According to the Organ Procurement and Transplantation Network (OPTN) and the Scientific Registry of Transplant Recipients (SRTR) report, ESW-based IS is utilized in 25% of KTRs [55]. ESW is generally considered for lower-immunologic-risk patients as opposed to high-risk, highly sensitized KTRs where glucocorticoids are usually continued long-term as part of their maintenance IS regimen.

A randomized, placebo-controlled trial compared ESW at day 7 after transplant to chronic glucocorticoid therapy [56]. No difference was observed in the primary composite endpoint of death, graft loss, or moderate to severe acute rejection between the two groups at 5 years [56]. Antibody-mediated rejection rate was similar, however mild biopsy-proven acute rejection (BPAR) was more common in the ESW arm. This was primarily driven by the induction type since the BPAR group was enriched in KTRs induced with IL-2 receptor blockers [31]. Triglyceride levels, weight gain and post-transplant diabetes mellitus (PTDM) favored ESW. These findings were replicated in a study that examined 15-year outcomes on a steroid-free regimen [57]. In living donor kidney recipients, ESW was linked to enhanced death-censored graft survival. Among deceased donor kidney recipients, ESW was associated with both improved patient survival and death-censored graft survival. Additionally, ESW recipients experienced lower rates of PTDM, cardiac complications, avascular necrosis, cytomegalovirus (CMV) infection, and cataracts [57].

In the INTAC study, about five hundred KTRs were randomized to alemtuzumab or conventional induction therapy (basiliximab or rabbit antithymocyte globulin (ATG) for high-risk patients) [58]. All patients received maintenance therapy with mycophenolic acid and tacrolimus, with ESW implemented by day five. The incidence of BPAR was lower in the alemtuzumab group compared to the conventional induction group (10% versus 22% at three years, *p* = 0.003). These findings favoring alemtuzumab were limited to the low immunologic risk group induced by basiliximab. When compared with ATG, alemtuzumab had similar efficacy [58]. The HARMONY study is an open-label, randomized controlled trial involving six hundred KTRs. It compared the effects of ESW in patients receiving either ATG or basiliximab induction to those who continued steroid therapy with basiliximab induction [59]. ATG induction was not associated with decreased BPAR, and patient and death censored graft survival were comparable at 1 year. The BPAR rate in the basiliximab groups was around 11% which is lower than the commonly cited rates in previous IL-2 receptor blocker studies [59]. The FREEDOM study included an arm which was completely steroid-free, in addition to ESW and steroid continuation arms. All patients received basiliximab induction [60]. The incidence of BPAR, graft loss, or mortality was highest in the steroid-free group, followed by the early steroid withdrawal and steroid maintenance groups [60]. In recipients undergoing re-transplantation, ESW was significantly associated with an increased risk of acute rejection (OR 1.42, *p* < 0.001) and graft failure (HR 1.24, *p* = 0.003) [61]. Overall, current evidence favors ESW in KTR undergoing lymphocyte-depleting induction, while its use with basiliximab induction warrants caution.

***Mammalian target of rapamycin inhibitor-based immunosuppression regimens:*** Mammalian target of rapamycin inhibitors (mTOR-i) are immunosuppressive medications that may spare or reduce CnI exposure [62,63,64,65,66,67,68,69,70]. The ELITE Symphony trial included a sirolimus-based arm which was inferior to the CnI arms [49]. Sirolimus was linked to higher BPAR rates, lower GFR, and reduced graft survival compared to CnI [49]. The ORION trial randomized patients to three regimens: sirolimus with tacrolimus followed by tacrolimus withdrawal at week 13, sirolimus with mycophenolic acid, or tacrolimus with mycophenolic acid [70]. The sirolimus plus mycophenolic acid group had the highest acute rejection rate at one year (31%), compared to 15% in the tacrolimus withdrawal group and 8% in the tacrolimus plus mycophenolic acid group [70]. Given the overall inferior outcomes of mTOR-i replacing CnI in IS regimens, subsequent studies primarily explored late CnI-to-mTOR-i conversion post-transplant or strategies combining low-dose CnI with mTOR-i [62,63,64,68,69].

In the ZEUS trial, KTRs were allocated to either a regimen comprising mycophenolic acid, cyclosporine, and prednisone or a similar regimen with planned cyclosporine-to-everolimus conversion at 4.5 months post-transplant [63,64]. At the 5-year follow-up, the cumulative incidence of BPAR was higher in the everolimus conversion arm (13.6%) compared to the cyclosporine maintenance group (7.5%), primarily attributed to Banff 1A-grade rejection [38]. Despite this, everolimus conversion was linked to superior kidney function at both 1 and 5 years [62,63]. Similarly, the ELEVATE trial examined 715 KTRs on mycophenolic acid, CnI, and prednisone, randomizing them between 10 and 14 weeks to either continue CnI therapy or switch to everolimus [68]. At 1 year, GFR changes were comparable between groups, though BPAR and anti-HLA class I DSA occurred more frequently in the everolimus arm. Additionally, treatment cessation resulting from adverse effects was markedly higher in the everolimus group (23.6% vs. 8.4% in the CnI arm) [68]. The CONVERT study, which randomized 830 patients at 6 months post-transplant to either sirolimus conversion or continued CnI therapy, found no significant difference in the proportion of patients achieving a GFR > 40 mL/min/1.73 m^2^ at 2 years based on an intent-to-treat analysis [65]. However, therapy-based evaluation suggested a GFR benefit with sirolimus conversion, particularly in those with baseline GFR > 40 mL/min/1.73 m^2^. Sirolimus conversion was associated with increased proteinuria, but BPAR rates, as well as graft and patient survival, remained comparable between groups. Notably, adverse events were more frequent in the sirolimus cohort, except for malignancy, which was lower in the sirolimus arm (3.8%) relative to the CnI continuation group (11%) [65].

The TRANSFROM study is a prospective, open label trial performed in 2000 lower-immunologic-risk KTRs. In this study, everolimus with reduced dose CnI regimen was compared to mycophenolic acid with standard dose CnI [62,69]. Patients received induction therapy with either basiliximab or ATG. In the reduced CnI dose cohort, everolimus trough levels were maintained between 3 and 8 ng/mL. Tacrolimus trough targets were stratified as 4–7 ng/mL for the initial 2 months, 2–5 ng/mL for months 3–6, and 2–4 ng/mL thereafter. Corresponding cyclosporine trough levels were set at 100–150, 50–100, and 25–50 ng/mL, respectively. In the mycophenolic acid cohort, tacrolimus trough targets were set at 8–12 ng/mL for the first 2 months, 6–10 ng/mL for months 3–6, and 5–8 ng/mL thereafter. Cyclosporine trough targets in this group were 200–300, 150–200, and 100–200 ng/mL, respectively. Steroid therapy was determined by individual center protocols, with a required prednisone minimum of 5 mg [62,69]. Everolimus combined with a reduced-dose CnI demonstrated non-inferiority compared to the standard regimen for the composite endpoint of BPAR or GFR < 50 mL/min/1.73 m^2^ at 2 years. The incidence of dnDSA was lower in the everolimus/reduced-dose CnI arm (12.3% vs. 17.6% in the standard regimen). Consistent with findings from the CONVERT study, treatment cessation due to adverse effects was more frequent in the everolimus group (27.2% vs. 15.0%). The rates of CMV and BK virus infection favored everolimus (2.8% versus 13.5% for CMV and 5.8% versus 10.3% for BK) [69].

In summary, mTORi-based regimens are associated with higher BPAR rate compared to CnI but less viral infections. Generally, they are not inferior when used with low-dose CnI in lower-immunologic risk-patients. mTORi should be used cautiously when the GFR is less than 40 mL/min/1.73 m^2^, and patients should be monitored for proteinuria.

***Belatacept-based immunosuppression regimens:*** Belatacept is a cytotoxic T-lymphocyte antigen 4 (CTLA-4) fusion protein which mitigates T-lymphocyte activation by binding to CD80/CD86 and inhibiting co-stimulation signal 2. It is approved for primary prevention of rejection and can be considered as an alternative for CnI.

The phase 3 trials BENEFIT and BENEFIT-EXT evaluated belatacept in comparison to cyclosporine among KTRs who received either standard or expanded criteria donor kidneys, respectively [71,72,73,74]. All patients received basiliximab induction but were placed on either mycophenolic acid/belatacept/prednisone or mycophenolic acid/cyclosporine/prednisone maintenance. Belatacept was associated with an increase in early acute cellular rejection, however with less DSA formation. Similar 1- and 3-year patient and graft survivals were observed [71,73]. These findings were reproduced in the BENEFIT-EXT cohort transplanted with expanded criteria kidneys [46,48]. Belatacept was associated with an improvement in the composite outcome of patient and graft survival after seven years of follow up [75,76]. Although this was primarily driven by graft survival, a patient survival trend favoring belatacept was observed. Seven-year GFR was superior in the patients receiving belatacept.

The BEST trial assigned KTRs to one of three regimens, alemtuzumab/belatacept, ATG/belatacept, or ATG/tacrolimus, with all participants also receiving mycophenolic acid and ESW [77]. The composite outcome, encompassing patient mortality, graft failure, or GFR falling below 45 mL/min/1.73 m^2^, showed no significant difference across the groups. Aligning with findings from the BENEFIT cohort, belatacept recipients exhibited an elevated ACR rate, though rates of antibody-mediated or mixed rejection remained comparable.

To reduce the early ACR rate observed in subjects receiving belatacept, Adams et al. investigated a protocol where tacrolimus was transiently introduced in addition to belatacept [78]. In a study involving 745 patients, belatacept therapy was supplemented with tacrolimus for three to nine months before being completely tapered. Initially, tacrolimus trough levels were maintained at a higher range but were progressively lowered to 3–5 ng/mL by the six-month mark. This strategy effectively reduced ACR rates while preserving belatacept’s long-term GFR benefits. Additionally, no significant differences were observed in the incidence of severe infections, including CMV and BK viremia. This is contrary to the study by Chavarot et al. which suggested an association between belatacept and CMV disease [79].

Budde et al. examined conversion from CnI-based maintenance IS to belatacept [80]. In their study, stable patients were randomized to stay on CnI or convert to belatacept 6 to 60 months post-transplantation. Converted patients were tapered off CnI one month after initiating belatacept. The belatacept group experienced more acute rejection episodes than the CnI continuation group (8% versus 4%) but developed less de novo DSA (1% versus 7%, respectively). Bertrand et al. conducted a retrospective analysis evaluating belatacept as a rescue therapy in patients exhibiting chronic vascular alterations, defined by a Banff cv (chronic vasculopathy) score of ≥2. [81]. Seventeen patients who were converted from CnI to belatacept were matched with a CnI continuation group. There was an increase in the GFR from 23.5 mL/min/1.73 m^2^ to 30.4 mL/min/1.73m^2^ in the conversion group which was sustained at the 1-year mark.

Overall, the compiled evidence indicates that belatacept-based regimens contribute to superior long-term graft survival and function. However, the increased incidence of early ACR has led to the adoption of strategies incorporating a temporary overlap of CnI with belatacept, followed by a gradual tapering approach.

## 4. Practical Considerations

The predominant IS regimen continues to incorporate mycophenolic acid and tacrolimus, with or without prednisone. Initial dosing is set at 1000 mg twice daily for mycophenolate mofetil and 720 mg twice daily for mycophenolate sodium, with adjustments based on tolerability. While mycophenolate sodium may offer a more favorable gastrointestinal side effect profile, both formulations have been linked to failure to thrive. In such cases, transitioning to azathioprine at 1–2 mg/kg daily may be considered as an alternative antimetabolite strategy. Another common indication to using azathioprine is pregnancy since mycophenolic acid is contraindicated due to increased risk of fetal malformations and miscarriage. With azathioprine, monitoring for bone marrow suppression would be prudent. Special attention should be paid to discontinuing allopurinol if a patient is on it before prescribing azathioprine. Allopurinol inhibits the metabolism of azathioprine and increases the levels of 6-mercaptopurine which may exacerbate cytopenias. In some centers, it is common practice to check thiopurine S-methyltransferase (TPMT) enzyme activity prior to starting azathioprine. This is to ensure normal enzyme activity and ability to appropriately inactivate azathioprine.

Tacrolimus trough levels are initially maintained at a higher range during the first month post-transplant and are then progressively lowered over time, ensuring they do not fall below 6 ng/mL. Patients experiencing neurological side effects from tacrolimus may be switched from short-acting to once-daily extended-release formulation, LCP-tacro (Envarsus). LCP-tacro pharmacokinetics allow more gradual absorption, avoiding the drug peaks associated with short-acting tacrolimus and improving tremors [82]. If a tacrolimus/mTOR-i combination is considered, regard should be given to the potential synergistic renal tubular epithelial cell toxicity since they are both metabolized by the cytochrome, CYP3A. Levels should not exceed 4-6 ng/mL for tacrolimus and 3-8 ng/mL for mTOR-i [83].

For patients unable to tolerate tacrolimus, cyclosporine serves as an alternative, with target trough levels maintained at 150–250 ng/mL during the initial three months and subsequently tapered to 100–150 ng/mL. Tacrolimus is primarily linked to metabolic complications such as PTDM, along with tremors and hair thinning, which frequently contribute to treatment discontinuation. In contrast, cyclosporine is associated with hirsutism and gingival hyperplasia. Both agents have been implicated in posterior reversible encephalopathy, a syndrome presenting with central nervous system disturbances, including headache, seizures, and altered mental status. Continued prednisone use is recommended in cases where native kidney disease results from glomerulonephritides, particularly IgA nephropathy, given its association with improved outcomes. Additionally, steroid maintenance is advised for KTRs with heightened immunologic risk and those requiring systemic corticosteroids for autoimmune conditions [84].

Transitioning to an mTOR-i regimen is advisable for patients with a history of non-melanomatous skin cancer, as it has been associated with a lower recurrence risk [85]. However, careful consideration is required in those with active wounds, given that mTOR-i can hinder wound healing and increase susceptibility to lymphocele formation [86,87]. mTOR-i should be avoided in KTRs with proteinuric disease since they may aggravate underlying podocytopathies [88,89].

Belatacept is approved for primary prevention of kidney transplant rejection in EBV seropositive patients. The exclusion of EBV-naive KTRs is due to the association observed between EBV seronegativity and post-transplant lymphoproliferative disease (PTLD), particularly central nervous system PTLD [90]. The broader adoption of belatacept has been somewhat restricted due to logistical barriers, including limited access to infusion centers in rural regions and a higher incidence of early ACR [91]. Additionally, definitive evidence on the ideal dosing frequency in KTRs experiencing infections or malignancies remains unavailable.

## 5. Future Directions

Advancements in immunosuppression over the years have transformed kidney transplantation from an experimental procedure between identical twins into the definitive therapy for ESKD. While significant progress has been made in reducing acute rejection, long-term graft survival continues to fall short of optimal outcomes compared to CnI. This can be partially attributed to IS therapies themselves. Short of inducing tolerance, the holy grail of transplantation has been effective immunosuppression with a favorable side effect profile. CTLA-4 is a global regulator of immune cells with a T-cell subset-specific function including regulatory T-cell (Treg) development and function [92]. As a result, inhibition of Tregs by CTLA-4 may facilitate rejection. To circumvent this effect, other therapeutic targets have been investigated. VEL-101 is a novel therapeutic that inhibits co-stimulation by directly blocking CD28. It is a pegylated monoclonal anti-CD28 antibody fragment that is being developed as a subcutaneous, self-administration medication. CD28 plays a role in T-cell activation whereas CTLA-4 plays a central role in regulating T-cell activation. Direct blockade of CD28 on T-cells will inhibit their stimulation while preserving the immunoregulatory function of CTLA-4 [93,94,95]. Early-phase human studies are currently underway to determine the safety, pharmacokinetics, and potential efficacy of VEL-101 [93,96].

Advancing the development of novel therapeutics requires the identification of measurable surrogates for long-term graft survival. To facilitate practical study design, IS trials have primarily assessed short-term endpoints such as acute rejection, GFR, and graft loss. Differences in study outcomes have largely been influenced by acute rejection rates, where CnI demonstrates superior efficacy but remains chronically nephrotoxic. iBOX, a predictive risk model, integrates dynamic variables including GFR, proteinuria, DSA, and histologic findings to estimate long-term graft failure risk [97]. The European Medical Agency has approved iBOX as a predictive composite biomarker, with recent endorsement from the Food and Drug Administration in the United States as well. Implementing iBOX as a clinical endpoint would mark a significant advancement in evaluating novel immunosuppressive strategies and their impact on prolonged graft survival.

## Figures and Tables

**Table 1:** Maintenance immunosuppressants.

	Mechanism of Action	Effect on T-Cell Activation	Adverse Events
Cyclosporine A	Calcineurin inhibitor	Prevents NFAT activation, reducing IL-2 production and therefore, T-cell activation and proliferation [15].	Hypertension [16] Nephrotoxicity [8,17] Neurotoxicity [18,19] Gingival hyperplasia [20] Hirsutism [21]
Tacrolimus	Calcineurin inhibitor	Prevents NFAT activation and subsequent T-cell activation and proliferation [22].	Hypertension [16] Nephrotoxicity [17] Neurotoxicity (more severe compared to Cyclosporine A) [19,23] Hair loss [24] Post-transplant diabetes mellitus [25]
Sirolimus	mTOR inhibitor	Impedes T-cell progression from G1 to S phase, inhibiting proliferation [26].	Hematologic effects (anemia, thrombocytopenia, leukopenia) [27,28] Impaired wound healing [29,30] Hyperlipidemia and hypertriglyceridemia [31] Stomatitis (oral ulcers) [32]
Everolimus	mTOR inhibitor	Impedes T-cell progression from G1 to S phase, inhibiting proliferation [33].	Hematologic effects (anemia, thrombocytopenia, leukopenia) [34] Impaired wound healing [29,30] Hyperlipidemia and hypertriglyceridemia [31] Stomatitis (oral ulcers) [32]
Azathioprine	Purine analogue	Impedes DNA replication and T-cell proliferation by inhibiting purine synthesis [35].	Gastrointestinal adverse effects (anorexia, nausea, and vomiting) [36] Dose-related marrow suppression [37,38]
Mycophenolic acid	IMPDH inhibitor	Blocks guanine nucleotide synthesis impairing proliferation of lymphocytes including T-cells [39].	Diarrhea, nausea, vomiting [40] High infection risk [41,42] Bone marrow suppression [42]
CTLA-4-Ig (Belatacept)	Competes with CD28for CD80/86 binding	Inhibits co-stimulatory signal, preventing full T-cell activation [43].	Increased risk of infections [44,45] EBV-associated PTLD [46]
VEL-101	Anti-CD28 monoclonal antibody fragment	Selectively inhibits CD28-mediated effector T-cell co-stimulation while preserving CTLA-4 function [47].	

Abbreviations; CTLA4: Cytotoxic T-Lymphocyte-Associated Protein 4; EBV: Epstein–Barr Virus; IL-2: Interleukin-2; IMPDH: Inosine Monophosphate Dehydrogenase; mTOR: Mammalian Target of Rapamycin; NFAT: Nuclear Factor of Activated T-Cells; PTLD: Post-Transplant Lymphoproliferative Disorder.

## Abbreviations

The following abbreviations are used in this manuscript:

ACR	Acute cellular rejection
APC	Antigen-presenting cell
BPAR	Biopsy-proven acute rejection
CMV	Cytomegalovirus
CnI	Calcineurin inhibitor
dnDSA	De novo donor-specific antibody
ESKD	End-stage kidney disease
ESW	Early steroid withdrawal
GFR	Glomerular filtration rate
IS	Immunosuppression
KTR	Kidney transplant recipient
mTOR	Mammalian target of rapamycin
mTOR-i	Mammalian target of rapamycin inhibitors
NFAT	Nuclear factor of activated T-cells
OPTN	Organ Procurement and Transplantation Network
PTDM	Post-transplant diabetes mellitus
PTLD	Post-transplant lymphoproliferative disease
SRTR	Scientific Registry of Transplant Recipients
TPMT	Thiopurine S-methyltransferase

## References

[1] Shi B., Ying T., Chadban S.J. (2023). Survival after kidney transplantation compared with ongoing dialysis for people over 70 years of age: A matched-pair analysis. Am. J. Transplant..

[2] de Boer S.E., Knobbe T.J., Kremer D., van Munster B.C., Nieuwenhuijs-Moeke G.J., Pol R.A., Bakker S.J., Berger S.P., Sanders J.S.F. (2024). Kidney Transplantation Improves Health-Related Quality of Life in Older Recipients. Transpl. Int..

[3] Ryu J.-H., Koo T.Y., Ro H., Cho J.-H., Kim M.-G., Huh K.H., Park J.B., Lee S., Han S., Kim J. (2021). Better health-related quality of life in kidney transplant patients compared to chronic kidney disease patients with similar renal function. PLoS ONE.

[4] Starzl T.E. (1994). The early days of transplantation. JAMA.

[5] Jing L., Yao L., Zhao M., Peng L.-P., Liu M. (2018). Organ preservation: From the past to the future. Acta Pharmacol. Sin..

[6] Colombo D., Ammirati E. (2011). Cyclosporine in transplantation—A history of converging timelines. J. Boil. Regul. Homeost. Agents.

[7] Tedesco D., Haragsim L. (2012). Cyclosporine: A Review. J. Transplant..

[8] Burdmann E.A., Andoh T.F., Yu L., Bennett W.M. (2003). Cyclosporine nephrotoxicity. Semin. Nephrol..

[9] Naesens M., Kuypers D.R., Sarwal M. (2009). Calcineurin inhibitor nephrotoxicity. Clin. J. Am. Soc. Nephrol..

[10] Clarkson M.R., Sayegh M.H. (2005). T-cell costimulatory pathways in allograft rejection and tolerance. Transplantation.

[11] Schwartz R.H. (1990). A cell culture model for T lymphocyte clonal anergy. Science.

[12] Hogan P.G. (2017). Calcium–NFAT transcriptional signalling in T cell activation and T cell exhaustion. Cell Calcium.

[13] Chi H. (2012). Regulation and function of mTOR signalling in T cell fate decisions. Nat. Rev. Immunol..

[14] Nayak S.P., Bagchi B., Roy S. (2022). Effects of immunosuppressants on T-cell dynamics: Understanding from a generic coarse-grained immune network model. J. Biosci..

[15] Schreiber S.L., Crabtree G.R. (1992). The mechanism of action of cyclosporin A and FK506. Immunol. Today.

[16] Hoorn E.J., Walsh S.B., McCormick J.A., Fürstenberg A., Yang C.-L., Roeschel T., Paliege A., Howie A.J., Conley J., Bachmann S. (2011). The calcineurin inhibitor tacrolimus activates the renal sodium chloride cotransporter to cause hypertension. Nat. Med..

[17] de Mattos A.M., Olyaei A.J., Bennett W.M. (2000). Nephrotoxicity of immunosuppressive drugs: Long-term consequences and challenges for the future. Am. J. Kidney Dis..

[18] Schwartz R.B., Bravo S.M., Klufas R.A., Hsu L., Barnes P.D., Robson C.D., Antin J.H. (1995). Cyclosporine neurotoxicity and its relationship to hypertensive encephalopathy: CT and MR findings in 16 cases. Am. J. Roentgenol..

[19] European FK506 Multicentre Liver Study Group (1994). Randomised trial comparing tacrolimus (FK506) and cyclosporin in prevention of liver allograft rejection. Lancet.

[20] Lauritano D., Palmieri A., Lucchese A., Di Stasio D., Moreo G., Carinci F. (2020). Role of Cyclosporine in Gingival Hyperplasia: An In Vitro Study on Gingival Fibroblasts. Int. J. Mol. Sci..

[21] Takahashi T., Kamimura A. (2001). Cyclosporin A promotes hair epithelial cell proliferation and modulates protein kinase C expression and translocation in hair epithelial cells. J. Investig. Dermatol..

[22] Thomson A.W., Bonham C.A., Zeevi A. (1995). Mode of action of tacrolimus (FK506): Molecular and cellular mechanisms. Ther. Drug Monit..

[23] Eidelman B.H., Abu-Elmagd K., Wilson J., Fung J.J., Alessiani M., Jain A., Takaya S., Todo S.N., Tzakis A., Van Thiel D. (1991). Neurologic complications of FK 506. Transplant. Proc..

[24] Shapiro R., Jordan M.L., Scantlebury V.P., Vivas C., McCauley J., Johnston J., Fung J.J., Starzl T.E. (1998). Alopecia as a consequence of tacrolimus therapy. Transplantation.

[25] Jindal R.M., Sidner R.A., Milgrom M.L. (1997). Post-transplant diabetes mellitus. The role of immunosuppression. Drug Saf..

[26] Sehgal S.N. (2003). Sirolimus: Its discovery, biological properties, and mechanism of action. Transplant. Proc..

[27] Hong J.C., Kahan B.D. (2000). Sirolimus-induced thrombocytopenia and leukopenia in renal transplant recipients: Risk factors, incidence, progression, and management12. Transplantation.

[28] Fishbane S., Cohen D.J., Coyne D.W., Djamali A., Singh A.K., Wish J.B. (2009). Posttransplant anemia: The role of sirolimus. Kidney Int..

[29] Dean P.G., Lund W.J., Larson T.S., Prieto M., Nyberg S.L., Ishitani M.B., Kremers W.K., Stegall M.D. (2004). Wound-healing complications after kidney transplantation: A prospective, randomized comparison of sirolimus and tacrolimus1. Transplantation.

[30] Mehrabi A., Fonouni H., Wente M., Sadeghi M., Eisenbach C., Encke J., Schmied B., Libicher M., Zeier M., Weitz J. (2006). Wound complications following kidney and liver transplantation. Clin. Transplant..

[31] Morrisett J.D., Abdel-Fattah G., Hoogeveen R., Mitchell E., Ballantyne C.M., Pownall H.J., Opekun A.R., Jaffe J.S., Oppermann S., Kahan B.D. (2002). Effects of sirolimus on plasma lipids, lipoprotein levels, and fatty acid metabolism in renal transplant patients. J. Lipid Res..

[32] Martins F., de Oliveira M.A., Wang Q., Sonis S., Gallottini M., George S., Treister N. (2013). A review of oral toxicity associated with mTOR inhibitor therapy in cancer patients. Oral Oncol..

[33] Houghton P.J. (2010). Everolimus. Clin. Cancer Res..

[34] Chen A., Chen L., Al-Qaisi A., Romond E., Awasthi M., Kadamyan-Melkumyan V., Massarweh S. (2015). Everolimus-induced hematologic changes in patients with metastatic breast cancer. Clin. Breast Cancer.

[35] Aarbakke J., Janka-Schaub G., Elion G.B. (1997). Thiopurine biology and pharmacology. Trends Pharmacol. Sci..

[36] Weinshilboum R., Sladek S. (1980). Mercaptopurine pharmacogenetics—Monogenic inheritance of erythrocyte thiopurine methyltransferase activity. Am. J. Hum. Genet..

[37] Varma P., Prasher P., Madan H., Yashpal B. (1996). Azathioprine induced bone marrow suppression in live related renal allograft recipients. Med. J. Armed. Forces India.

[38] Connell W.R., Kamm M.A., Ritchie J.K., Lennard-Jones J.E. (1993). Bone marrow toxicity caused by azathioprine in inflammatory bowel disease: 27 years of experience. Gut.

[39] Allison A.C., Eugui E.M. (2000). Mycophenolate mofetil and its mechanisms of action. Immunopharmacology.

[40] Davies N.M., Grinyó J., Heading R., Maes B., Meier-Kriesche H.-U., Oellerich M. (2007). Gastrointestinal side effects of mycophenolic acid in renal transplant patients: A reappraisal. Nephrol. Dial. Transplant..

[41] Bernabeu-Wittel M., Naranjo M., Cisneros J., Cañas E., Gentil M., Algarra G., Pereira P., González-Roncero F., de Alarcón A., Pachón J. (2002). Infections in renal transplant recipients receiving mycophenolate versus azathioprine-based immunosuppression. Eur. J. Clin. Microbiol. Infect. Dis..

[42] Datrino L.N., Boccuzzi M.L., Silva R.M., Castilho P.H.B.T., Riva W.J., Rocha J.S., Tustumi F. (2024). Safety and Efficacy of Mycophenolate Mofetil Associated With Tacrolimus for Kidney-pancreas and Kidney Transplantation: A Systematic Review and Meta-Analysis of Randomized Studies. Transplant. Proc..

[43] Belatacept. https://go.drugbank.com/drugs/DB06681.

[44] Marvin J.E., Azar M.M., Belfield K.D., Do V., Formica R., Cohen E.A. (2022). Overall Infectious Complications Related to Belatacept Conversion in Comparison to Tacrolimus in Kidney Transplant Recipients. Prog. Transplant..

[45] Terrec F., Jouve T., Malvezzi P., Janbon B., Bennani H.N., Rostaing L., Noble J. (2021). Belatacept Use after Kidney Transplantation and Its Effects on Risk of Infection and COVID-19 Vaccine Response. J. Clin. Med..

[46] Cherikh W.S., Kou T.D., Foutz J., Baker T.J., Gomez-Caminero A. (2025). Patterns of belatacept use and risk of post-transplant lymphoproliferative disorder in US kidney transplant recipients: An analysis of the Organ Procurement and Transplantation Network database. PLoS ONE.

[47] FR104/VEL-101. https://www.ose-immuno.com/en/our-products/fr104-modular/.

[48] Lentine K.L., Smith J.M., Hart A., Miller J., Skeans M.A., Larkin L., Robinson A., Gauntt K., Israni A.K., Hirose R. (2022). OPTN/SRTR 2020 Annual Data Report: Kidney. Am. J. Transplant..

[49] Ekberg H., Tedesco-Silva H., Demirbas A., Vítko Š., Nashan B., Gürkan A., Margreiter R., Hugo C., Grinyó J.M., Frei U. (2007). Reduced exposure to calcineurin inhibitors in renal transplantation. N. Engl. J. Med..

[50] Davis S., Gralla J., Klem P., Tong S., Wedermyer G., Freed B., Wiseman A., Cooper J.E. (2018). Lower tacrolimus exposure and time in therapeutic range increase the risk of de novo donor-specific antibodies in the first year of kidney transplantation. Am. J. Transplant..

[51] Wiebe C., Gibson I.W., Blydt-Hansen T.D., Pochinco D., Birk P.E., Ho J., Karpinski M., Goldberg A., Storsley L., Rush D.N. (2015). Rates and determinants of progression to graft failure in kidney allograft recipients with de novo donor-specific Antibody. Am. J. Transplant..

[52] Wojciechowski D., Wiseman A. (2021). Long-Term Immunosuppression Management: Opportunities and Uncertainties. Clin. J. Am. Soc. Nephrol..

[53] Knight S.R., Russell N.K., Barcena L., Morris P.J. (2009). Mycophenolate mofetil decreases acute rejection and may improve graft survival in renal transplant recipients when compared with azathioprine: A systematic review. Transplantation.

[54] Gaston R.S., Kaplan B., Shah T., Cibrik D., Shaw L.M., Angelis M., Mulgaonkar S., Meier-Kriesche H.-U., Patel D., Bloom R.D. (2009). Fixed- or controlled-dose mycophenolate mofetil with standard- or reduced-dose calcineurin inhibitors: The Opticept trial. Am. J. Transplant..

[55] Lentine K.L., Smith J.M., Miller J.M., Bradbrook K., Larkin L., Weiss S., Handarova D.K., Temple K., Israni A.K., Snyder J.J. (2023). OPTN/SRTR 2021 Annual Data Report: Kidney. Am. J. Transplant..

[56] Woodle E.S., First M.R., Pirsch J., Shihab F., Gaber A.O., Van Veldhuisen P., Corticosteroid Withdrawal Study Group (2008). A prospective, randomized, double-blind, placebo-controlled multicenter trial comparing early (7 day) corticosteroid cessation versus long-term, low-dose corticosteroid therapy. Ann. Surg..

[57] Serrano O.K., Kandaswamy R., Gillingham K., Chinnakotla S., Dunn T.B., Finger E., Payne W., Ibrahim H., Kukla A., Spong R. (2017). Rapid Discontinuation of Prednisone in Kidney Transplant Recipients: 15-Year Outcomes From the University of Minnesota. Transplantation.

[58] Hanaway M.J., Woodle E.S., Mulgaonkar S., Peddi V.R., Kaufman D.B., First M.R., Croy R., Holman J. (2011). Alemtuzumab induction in renal transplantation. N. Engl. J. Med..

[59] Thomusch O., Wiesener M., Opgenoorth M., Pascher A., Woitas R.P., Witzke O., Jaenigen B., Rentsch M., Wolters H., Rath T. (2016). Rabbit-ATG or basiliximab induction for rapid steroid withdrawal after renal transplantation (Harmony): An open-label, multicentre, randomised controlled trial. Lancet.

[60] Vincenti F., Schena F.P., Paraskevas S., Hauser I.A., Walker R.G., Grinyo J. (2008). A Randomized, multicenter study of steroid avoidance, early steroid withdrawal or standard steroid therapy in kidney transplant recipients. Am. J. Transplant..

[61] Bae S., Chen Y., Sandal S., Lentine K.L., Schnitzler M., Segev D.L., DeMarco M.A.M. (2024). Association of early steroid withdrawal with kidney transplant outcomes in first-transplant and retransplant recipients. Nephrol Dial Transplant..

[62] Pascual J., Berger S.P., Witzke O., Tedesco H., Mulgaonkar S., Qazi Y., Chadban S., Oppenheimer F., Sommerer C., Oberbauer R. (2018). Everolimus with Reduced Calcineurin Inhibitor Exposure in Renal Transplantation. J. Am. Soc. Nephrol..

[63] Budde K., Becker T., Arns W., Sommerer C., Reinke P., Eisenberger U., Kramer S., Fischer W., Gschaidmeier H., Pietruck F. (2011). Everolimus-based, calcineurin-inhibitor-free regimen in recipients of de-novo kidney transplants: An open-label, randomised, controlled trial. Lancet.

[64] Budde K., Lehner F., Sommerer C., Reinke P., Arns W., Eisenberger U., Wüthrich R.P., Mühlfeld A., Heller K., Porstner M. (2015). Five-year outcomes in kidney transplant patients converted from cyclosporine to everolimus: The randomized ZEUS study. Am. J. Transplant..

[65] Schena F.P., Pascoe M.D., Alberu J., Rial M.d.C., Oberbauer R., Brennan D.C., Campistol J.M., Racusen L., Polinsky M.S., Goldberg-Alberts R. (2009). Conversion from calcineurin inhibitors to sirolimus maintenance therapy in renal allograft recipients: 24-month efficacy and safety results from the CONVERT trial. Transplantation.

[66] Shihab F., Qazi Y., Mulgaonkar S., McCague K., Patel D., Peddi V.R., Shaffer D. (2017). Association of Clinical Events With Everolimus Exposure in Kidney Transplant Patients Receiving Low Doses of Tacrolimus. Am. J. Transplant..

[67] Qazi Y., Shaffer D., Kaplan B., Kim D.Y., Luan F.L., Peddi V.R., Shihab F., Tomlanovich S., Yilmaz S., McCague K. (2017). Efficacy and Safety of Everolimus Plus Low-Dose Tacrolimus Versus Mycophenolate Mofetil Plus Standard-Dose Tacrolimus in De Novo Renal Transplant Recipients: 12-Month Data. Am. J. Transplant..

[68] de Fijter J.W., Holdaas H., Øyen O., Sanders J., Sundar S., Bemelman F.J., Sommerer C., Pascual J., Avihingsanon Y., Pongskul C. (2017). Early Conversion From Calcineurin Inhibitor- to Everolimus-Based Therapy Following Kidney Transplantation: Results of the Randomized ELEVATE Trial. Am. J. Transplant..

[69] Berger S.P., Sommerer C., Witzke O., Tedesco H., Chadban S., Mulgaonkar S., Qazi Y., de Fijter J.W., Oppenheimer F., Cruzado J.M. (2019). Two-year outcomes in de novo renal transplant recipients receiving everolimus-facilitated calcineurin inhibitor reduction regimen from the TRANSFORM study. Am. J. Transplant..

[70] Flechner S.M., Glyda M., Cockfield S., Grinyó J., Legendre C., Russ G., Steinberg S., Wissing K.M., Tai S.S. (2011). The ORION study: Comparison of two sirolimus-based regimens versus tacrolimus and mycophenolate mofetil in renal allograft recipients. Am. J. Transplant..

[71] Vincenti F., Charpentier B., Vanrenterghem Y., Rostaing L., Bresnahan B., Darji P., Massari P., Mondragon-Ramirez G., Agarwal M., Di Russo G. (2010). A Phase III study of belatacept-based immunosuppression regimens versus cyclosporine in renal transplant recipients (benefit study). Am. J. Transplant..

[72] Durrbach A., Pestana J.M., Pearson T., Vincenti F., Garcia V.D., Campistol J., del Carmen Rial M., Florman S., Block A., Di Russo G. (2010). A Phase III Study of Belatacept Versus Cyclosporine in Kidney Transplants from Extended Criteria Donors (BENEFIT-EXT Study). Am. J. Transplant..

[73] Vincenti F., Larsen C.P., Alberu J., Bresnahan B., Garcia V.D., Kothari J., Lang P., Urrea E.M., Massari P., Mondragon-Ramirez G. (2012). Three-Year Outcomes from BENEFIT, a Randomized, Active-Controlled, Parallel-Group Study in Adult Kidney Transplant Recipients. Am. J. Transplant..

[74] Pestana J.O.M., Grinyo J.M., Vanrenterghem Y., Becker T., Campistol J.M., Florman S., Garcia V.D., Kamar N., Lang P., Manfro R.C. (2012). Three-year outcomes from BENEFIT-EXT: A phase III study of belatacept versus cyclosporine in recipients of extended criteria donor kidneys. Am. J. Transplant..

[75] Vincenti F., Rostaing L., Grinyo J., Rice K., Steinberg S., Gaite L., Moal M.C., Mondragon-Ramirez G.A., Kothari J., Polinsky M.S. (2016). Belatacept and Long-Term Outcomes in Kidney Transplantation. N. Engl. J. Med..

[76] Durrbach A., Pestana J.M., Florman S., Del Carmen Rial M., Rostaing L., Kuypers D., Matas A., Wekerle T., Polinsky M., Meier-Kriesche H.U. (2016). Long-Term Outcomes in Belatacept-Versus Cyclosporine-Treated Recipients of Extended Criteria Donor Kidneys: Final Results From BENEFIT-EXT, a Phase III Randomized Study. Am. J. Transplant..

[77] Woodle E.S., Kaufman D.B., Shields A.R., Leone J., Matas A., Wiseman A., West-Thielke P., Sa T., King E.C., Alloway R.R. (2020). Belatacept-based immunosuppression with simultaneous calcineurin inhibitor avoidance and early corticosteroid withdrawal: A prospective, randomized multicenter trial. Am. J. Transplant..

[78] Adams A.B., Goldstein J., Garrett C., Zhang R., Patzer R.E., Newell K.A., Turgeon N.A., Chami A.S., Guasch A., Kirk A.D. (2017). Belatacept Combined With Transient Calcineurin Inhibitor Therapy Prevents Rejection and Promotes Improved Long-Term Renal Allograft Function. Am. J. Transplant..

[79] Chavarot N., Divard G., Scemla A., Amrouche L., Aubert O., Leruez-Ville M., Timsit M.O., Tinel C., Zuber J., Legendre C. (2021). Increased incidence and unusual presentations of CMV disease in kidney transplant recipients after conversion to belatacept. Am. J. Transplant..

[80] Budde K., Prashar R., Haller H., Rial M.C., Kamar N., Agarwal A., de Fijter J.W., Rostaing L., Berger S.P., Djamali A. (2021). Conversion from Calcineurin Inhibitor– to Belatacept-Based Maintenance Immunosuppression in Renal Transplant Recipients: A Randomized Phase 3b Trial. J. Am. Soc. Nephrol..

[81] Bertrand D., Cheddani L., Etienne I., François A., Hanoy M., Laurent C., Lebourg L., Le Roy F., Lelandais L., Loron M. (2017). Belatacept Rescue Therapy in Kidney Transplant Recipients With Vascular Lesions: A Case Control Study. Am. J. Transplant..

[82] Langone A., Steinberg S.M., Gedaly R., Chan L.K., Shah T., Sethi K.D., Nigro V., Morgan J.C., Formica R.N., STRATO Investigators (2015). Switching STudy of Kidney TRansplant PAtients with Tremor to LCP-TacrO (STRATO): An open-label, multicenter, prospective phase 3b study. Clin. Transplant..

[83] Andoh T.F., Lindsley J., Franceschini N., Bennett W.M. (1996). Synergistic effects of cyclosporine and rapamycin in a chronic nephrotoxicity model1. Transplantation.

[84] Clayton P., McDonald S., Chadban S. (2011). Steroids and recurrent IgA nephropathy after kidney transplantation. Am. J. Transplant..

[85] Campbell S.B., Walker R., Tai S.S., Jiang Q., Russ G.R. (2012). Randomized controlled trial of sirolimus for renal transplant recipients at high risk for nonmelanoma skin cancer. Am. J. Transplant..

[86] Valente J.F., Hricik D., Weigel K., Seaman D., Knauss T., Siegel C.T., Bodziak K., Schulak J.A. (2003). Comparison of sirolimus vs. mycophenolate mofetil on surgical complications and wound healing in adult kidney transplantation. Am. J. Transplant..

[87] Langer R.M., Kahan B.D. (2002). Incidence, therapy, and consequences of lymphocele after sirolimus-cyclosporine-prednisone immunosuppression in renal transplant recipients1. Transplantation.

[88] Letavernier E., Bruneval P., Vandermeersch S., Perez J., Mandet C., Belair M.-F., Haymann J.-P., Legendre C., Baud L. (2009). Sirolimus interacts with pathways essential for podocyte integrity. Nephrol. Dial. Transplant..

[89] Kopp J.B., Anders H.J., Susztak K., Podestà M.A., Remuzzi G., Hildebrandt F., Romagnani P. (2020). Podocytopathies. Nat. Rev. Dis. Primers.

[90] Grinyó J., Charpentier B., Pestana J.M., Vanrenterghem Y., Vincenti F., Reyes-Acevedo R., Apanovitch A.M., Gujrathi S., Agarwal M., Thomas D. (2010). An integrated safety profile analysis of belatacept in kidney transplant recipients. Transplantation.

[91] Vincenti F. (2017). Belatacept: The challenges with transformational drugs. Transl. Androl. Urol..

[92] Krummey S.M., Ford M.L. (2014). Braking bad: Novel mechanisms of CTLA-4 inhibition of T cell responses. Am. J. Transplant..

[93] Poirier N., Blancho G., Hiance M., Mary C., Van Assche T., Lempoels J., Ramael S., Wang W., Thepenier V., Braudeau C. (2016). First-in-Human Study in Healthy Subjects with FR104, a Pegylated Monoclonal Antibody Fragment Antagonist of CD28. J. Immunol..

[94] Poirier N., Mary C., Dilek N., Hervouet J., Minault D., Blancho G., Vanhove B. (2012). Preclinical efficacy and immunological safety of FR104, an antagonist anti-CD28 monovalent Fab′ antibody. Am. J. Transplant..

[95] Poirier N., Dilek N., Mary C., Ville S., Coulon F., Branchereau J., Tillou X., Charpy V., Pengam S., Nerriere-Daguin V. (2015). FR104, an antagonist anti-CD28 monovalent Fab’ antibody, prevents alloimmunization and allows calcineurin inhibitor minimization in nonhuman primate renal allograft. Am. J. Transplant..

[96] (2022). A Phase 1, Randomized, Double Blind, Placebo Controlled, Dose Escalation Study to Evaluate the Safety, Tolerability, Pharmacokinetics, and Pharmacodynamics of VEL-101 Administered Intravenously or Subcutaneously in Healthy Subjects. https://adisinsight.springer.com/trials/700348143.

[97] Rampersad C., Bau J.T., Orchanian-Cheff A., Kim S.J. (2025). iBox as a Predictor of Kidney Allograft Failure: A Systematic Review. Am. J. Kidney Dis..

